# Atypical Location of Lichen Simplex Chronicus

**DOI:** 10.7759/cureus.79653

**Published:** 2025-02-25

**Authors:** Dana Simon, Mark Schury

**Affiliations:** 1 College of Osteopathic Medicine, Michigan State University College of Osteopathic Medicine, East Lansing, USA; 2 Department of Family Medicine, McLaren Oakland Hospital, Pontiac, USA

**Keywords:** chronic itch, chronic lichenified eczema, lichen simplex chronicus, psoriasiform dermatitis, severe pruritus

## Abstract

Lichen simplex chronicus (LSC) is a chronic itch condition, severely impacting many patients’ quality of life. The burden of this condition has been referred to as debilitating, which can lead to a sequela of chronic anxiety, depression, and stress. LSC is characterized by intractable scratching and manipulation of the skin, resulting in symmetrically thickened, scaly plaques. LSC is extremely common and is one of the most common dermatological chief complaints in clinics. This case report highlights a 68-year-old male patient presenting for an evaluation of eczema on his back associated with unrelenting pruritus in a location that he could not reach, ultimately diagnosed as LSC upon histological evaluation. Patients can experience severe suffering from chronic itching, often compared to that of chronic pain conditions. Because of this, this case emphasizes the importance of prompt diagnosis and treatment and to suspect LSC in hyperpigmented pruritic plaques, regardless of bodily location.

## Introduction

Lichen simplex chronicus (LSC) is a chronic itch condition, severely impacting many patients’ quality of life, often leading to anxiety, depression, and increased stress. Constant scratching, rubbing, and manipulating the skin leads to LSC, resulting in thick and scaly plaques. These plaques can also have areas of lighter pigmentation, often a post-inflammatory reaction to the chronic scratching of the lesion. They are often symmetric and well-circumscribed. Psychiatric disorders and environmental changes can lead to primary LSC, but more commonly, it is secondary to cutaneous conditions such as eczema or psoriasis [1]. This highlights the importance of treating such conditions early and adequately before they become chronic conditions like LSC. It is most common in women, and the peak incidence is between 30 and 50 years of age [2]. It classically presents in the easily self-accessible areas of the body such as the scalp, forearm, extensors, hands, and genitals. LSC is extremely common, and one of the most common dermatological chief complaints in clinics. Its prevalence is estimated to be 12%. Differential diagnoses are psoriasis, eczema, squamous cell carcinoma of the skin, and hypertrophic lichen planus. Potential tests consist of skin biopsy, patch testing, fungal culture, skin scrapings, and blood work. Evaluating the underlying cause of severe pruritus is important, as prompt adequate treatment helps improve patient satisfaction and well-being. This case report highlights a 68-year-old male patient presenting for an evaluation of eczema on his back associated with unrelenting pruritus in a location that he could not reach, ultimately diagnosed as LSC upon histological evaluation.

## Case presentation

A 68-year-old male patient presented for an eczema evaluation on his back due to severe pruritus. He stated it had been there for as long as he could remember, and he had not been able to apply any topical medication as his hands did not reach the affected location on the back. His medical history consists of hyperlipidemia, type 2 diabetes mellitus, and hypertension. Medications include atorvastatin, baby aspirin, and metformin. 

**Figure 1:**
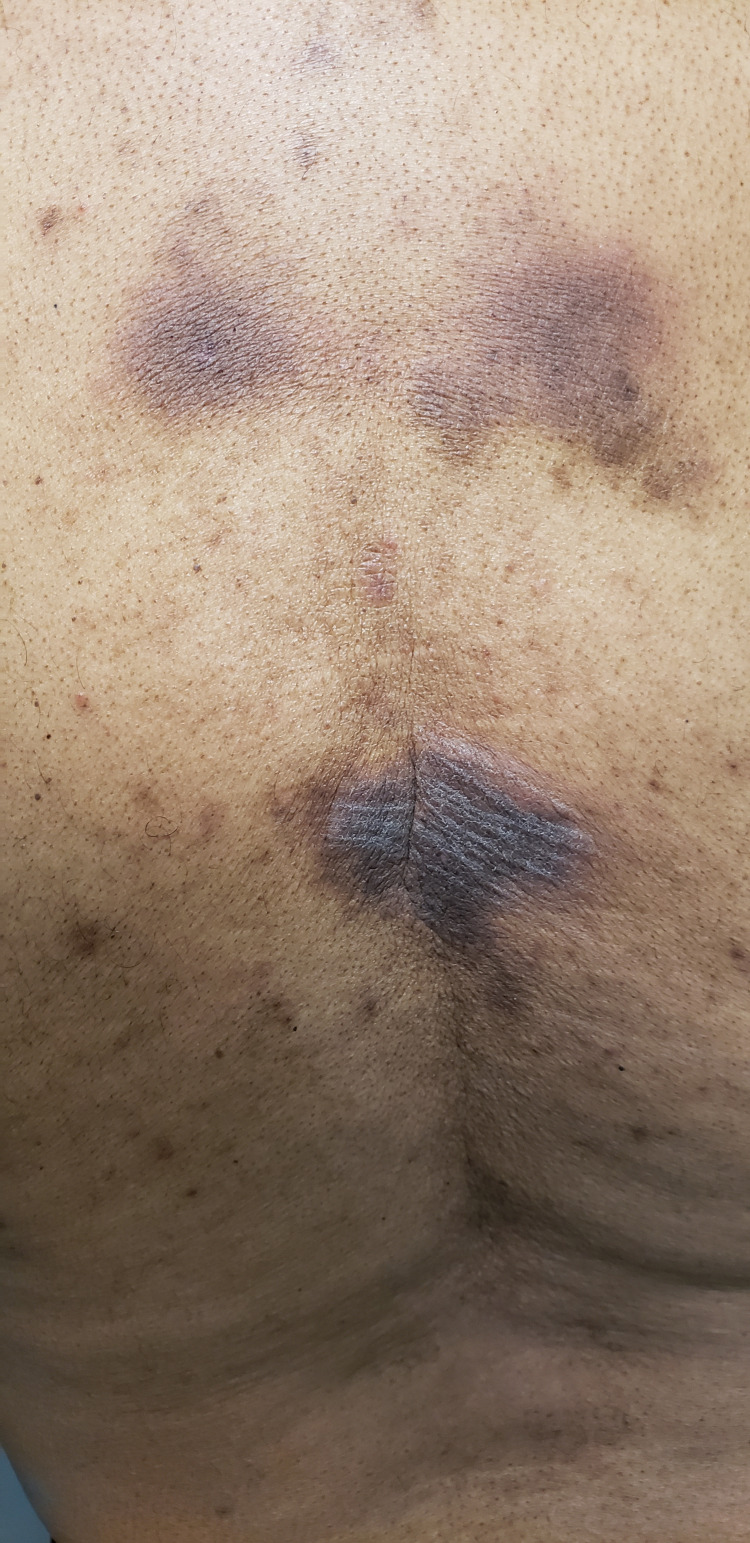
Three plaques on the midback, violaceous in color

**Figure 2:**
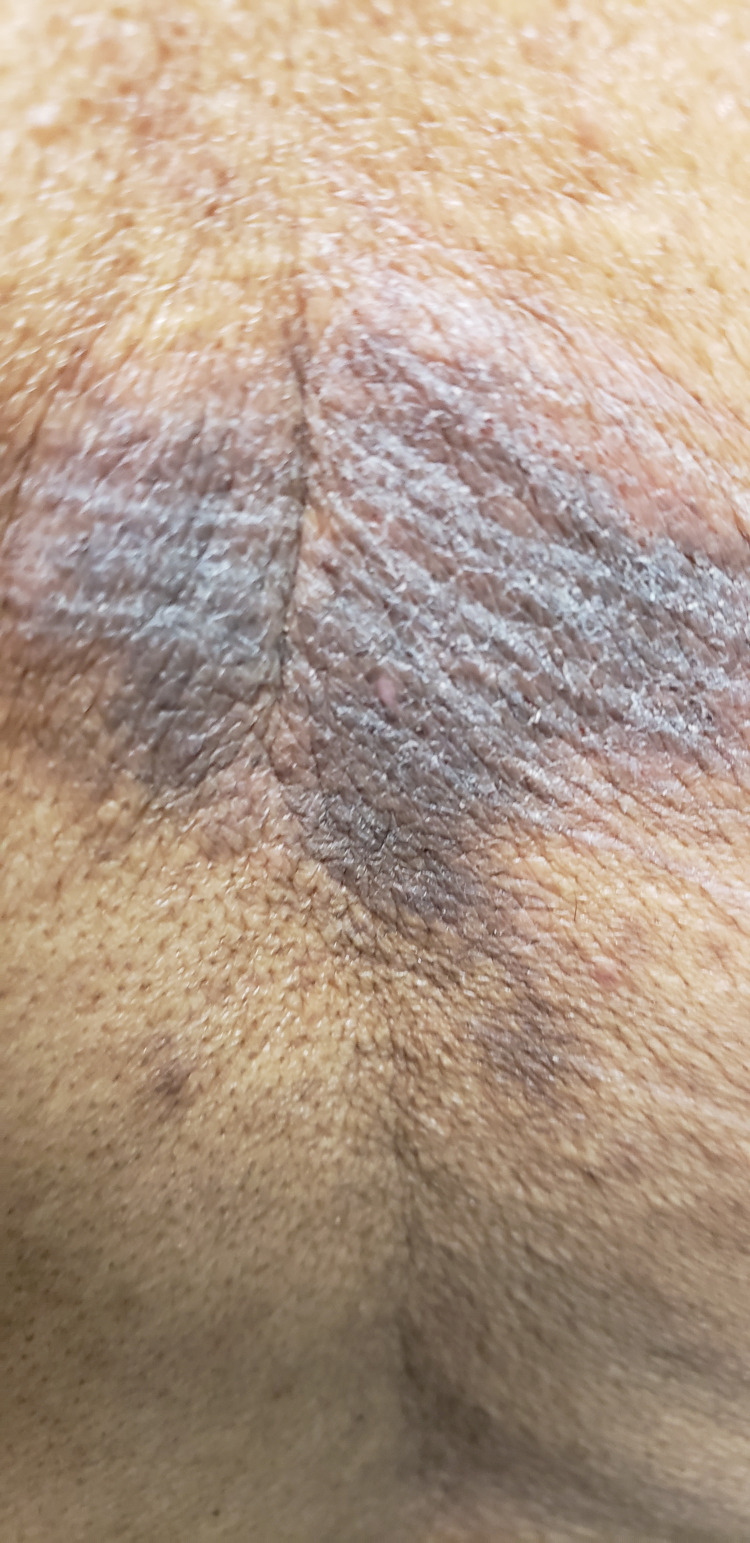
Lichenified and violaceous plaque

On the physical exam, blood pressure was 137/88, and BMI was 31.28. Integumentary findings consisted of three plaques on the midback (Figure 1), which were violaceous in color with some lichenification (Figure 2). It was nonblanching, painless, and unchanged over the years. The remainder of the physical exam was unremarkable and noncontributory. 

Diagnostic assessment included punch biopsy, in which a 3.00 mm biopsy was taken from the left upper plaque, and a second 3.00 punch biopsy was taken from the lower plaque. Histological evaluation showed epidermal psoriasiform dermatitis and was consistent with eczematous type dermatitis as well as LSC. There was no concern of malignancy, and it was periodic acid-Schiff (PAS) stain negative for fungus. 

**Figure 3:**
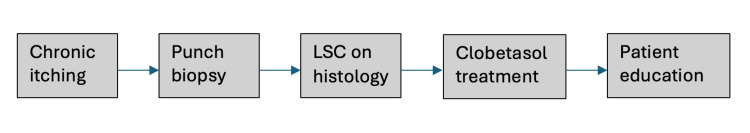
Chronology of events and management of the patient's presentation LSC: Lichen simplex chronicus

The patient was advised to use clobetasol 0.05% topical cream, twice daily, on the affected areas. He received education on having his family members help him apply the cream, as he is unable to reach the affected locations. He was advised to follow up if his family notices ulceration, rapid changes in skin color, or unusual bleeding in the affected areas (Figure 3).

## Discussion

LSC is difficult to manage in that psychological comorbidities either preceding or as a result of the unrelenting itching may present. The first-line treatment of LSC is topical corticosteroids and antihistamines. If the symptoms of LSC are unresponsive to an initial trial of corticosteroids, occlusive dressing may be used on top of the corticosteroid, but this should be done sparingly, with a maximum of one hour per day and for now more than two weeks to avoid corticosteroid adverse effects [3]. As the condition improves, topical corticosteroids should be used less frequently to avoid adverse effects. If long-term treatment is necessary, a topical calcineurin inhibitor should be used in place of a corticosteroid [4]. 

LSC develops from what is known as the itch and scratch cycle. The pathophysiology is believed to involve unknown pruritogens, most likely nonhistaminergic due to its chronic process, which bind to C-nerve fibers, and then transmit a signal to the central nervous system. If the theory is correct in being nonhistaminergic, it involves pruritogens binding to transient receptor potential channels, which may respond to capsaicin [5]. This cycle is repeated, increasing pruritic sensitivity each time, which is the underlying mechanism of chronic unrelenting itch. Over time, the disruption of the skin barrier in LSC can lead to increased susceptibility to infection as well as oncogenesis.

A primary dermatosis such as eczematous type dermatitis, as seen in our patient, often leads to secondary LSC. Our case is unique in that our patient denied conscious rubbing or scratching of his affected lesions, which is the only known cause of histologically confirmed LSC. This raises the importance of the psychological component of unconsciously rubbing against surfaces or using various items to satisfy the urge to itch or rub a chronically pruritic lesion. LSC is typically only included in a differential in locations that are easy to reach on the body, which emphasizes the rarity of this case. Because of the rare possibility of malignant transformation of epithelial tissue in LSC, it is important to avoid ruling out a diagnosis of LSC based on the presumption that the affected area could not have been reached, and therefore not rubbed by the patient. Other conditions that can lead to chronic itching, and therefore LSC, are renal failure, biliary disease, polycythemia rubra vera, and gluten-sensitive enteropathy [6]. 

## Conclusions

This patient’s presentation highlights an atypical and unexpected location of LSC and a unique scenario of the patient being unaware of the chronic rubbing and manipulation of the affected area. LSC plaques typically present in easily accessible bodily areas, as the pathogenesis has to do with chronic scratching. Patients can experience severe suffering from chronic itching. Because of this, this case emphasizes the importance of prompt diagnosis and treatment and to suspect LSC in hyperpigmented pruritic plaques, regardless of bodily location.
